# Continuous Gradient Temperature Raman Spectroscopy of Fish Oils Provides Detailed Vibrational Analysis and Rapid, Nondestructive Graphical Product Authentication

**DOI:** 10.3390/molecules23123293

**Published:** 2018-12-12

**Authors:** C. Leigh Broadhurst, Walter F. Schmidt, Jianwei Qin, Kuanglin Chao, Moon S. Kim

**Affiliations:** 1 Sensors Development Laboratory, Environmental Microbial and Food Safety Laboratory, United States Department of Agriculture Agricultural Research Service, 10300 Baltimore Avenue, Beltsville, MD 20705, USA; walter.schmidt@ars.usda.gov (W.F.S.); jianwei.qin@ars.usda.gov (J.Q.); kevin.chao@ars.usda.gov (K.C.); moon.kim@ars.usda.gov (M.S.K.); 2 Department of Mechanical Engineering, University of Maryland Baltimore County, Baltimore, MD 21250, USA

**Keywords:** gradient temperature Raman spectroscopy, Raman spectroscopy, long chain polyunsaturated fatty acids, DHA, EPA, fish oil, cod liver oil, eicosapentaenoic acid, docosahexaenoic acid

## Abstract

**Background**: Gradient temperature Raman spectroscopy (GTRS) applies the continuous temperature gradients utilized in differential scanning calorimetry (DSC) to Raman spectroscopy, providing a new means for rapid high throughput material identification and quality control. **Methods**: Using 20 Mb three-dimensional data arrays with 0.2 °C increments and first/second derivatives allows complete assignment of solid, liquid and transition state vibrational modes. The entire set or any subset of the any of the contour plots, first derivatives or second derivatives can be utilized to create a graphical standard to quickly authenticate a given source. In addition, a temperature range can be specified that maximizes information content. **Results**: We compared GTRS and DSC data for five commercial fish oils that are excellent sources of docosahexaenoic acid (DHA; 22:6n-3) and eicosapentaenoic acid (EPA; 20:5n-3). Each product has a unique, distinctive response to the thermal gradient, which graphically and spectroscopically differentiates them. We also present detailed Raman data and full vibrational mode assignments for EPA and DHA. **Conclusion**: Complex lipids with a variety of fatty acids and isomers have three dimensional structures based mainly on how structurally similar sites pack. Any localized non-uniformity in packing results in discrete “fingerprint” molecular sites due to increased elasticity and decreased torsion.

## 1. Introduction

The absolute requirement for docosahexaenoic acid (DHA, 22:6n-3) in fast signal processing tissues including neuronal, retinal and cardiac is one of the most intriguing aspects of lipid biochemistry. Despite over 600 million years of evolution, the difference of just a single diene group has not been overcome: DHA is utilized exclusively from the diatom photoreceptor to the cephalopod eye to the primate brain [1,2,3,4,5,6,7]. The severe neural and visual functional deficits arising from the replacement of DHA with either docosapentaenoic acid (DPA 22:5n-3 or 22:5n-6) indicate that significant structural changes to lipid bilayers must occur [1]. With extreme n-3 polyunsaturated fatty acid (PUFA) deprivation, n-6DPA is incorporated into neural and retinal tissues leading to developmental disorders, neurodegeneration, dementia, memory loss, subnormal retinal signaling and loss of visual acuity [8,9,10,11].

Conventional infrared (FTIR) and Raman vibrational analysis has value for many applications, but does not provide any information indicating why DHA, DPA and eicosapentaenoic acid (EPA 20:5 n-3) are so vastly different biochemically. To the first order, all the long-chain PUFAs appear nearly identical, with the vibrational modes of most moieties degenerate, redundant or very broad [12,13,14,15,16]. We have developed continuous gradient temperature Raman spectroscopy (GTRS), which is a simple, rapid technique for determining the unique structures of the unsaturated fatty acids [17,18,19,20,21]. GTRS applies the temperature gradients utilized in differential scanning calorimetry (DSC) to Raman spectroscopy, providing a straightforward technique to identify molecular rearrangements that occur near and at phase transitions. GTRS can identify and differentiate specific carbon chain sites, allowing Raman analysis to explain why the long-chain PUFA exhibit such extreme functional differences. 

Collecting spectra in a temperature gradient provides information not readily available in constant temperature measurements, because the dynamic nature of the vibrational structure can be observed in real time as the molecule changes conformation. Therefore, GTRS allows for simple, graphical identification of even complex lipid mixtures, foods or lipid-containing matrices, and has potential uses for nondestructive high-throughput food, dietary supplement and pharmaceutical raw material identification. If it is determined that a sample varies from the standard beyond the specified limits, more precise but also far more expensive and time consuming analyses can be performed [22,23,24,25,26]. Previous authors have clearly shown that FTIR and Raman data calibrated with gas chromatography can provide accurate, rapid quantitative analyses of fatty acid species in fish oil, dramatically reducing analytical time and cost [13,15,26].

In this contribution we compare and analyze GTRS and DSC data for five commercial fish oils that are considered excellent sources of DHA and EPA. In addition, improved and extensive Raman data and vibrational mode assignments for EPA are presented and compared with previously published data for DHA. 

## 2. Results

**Figure 1:**
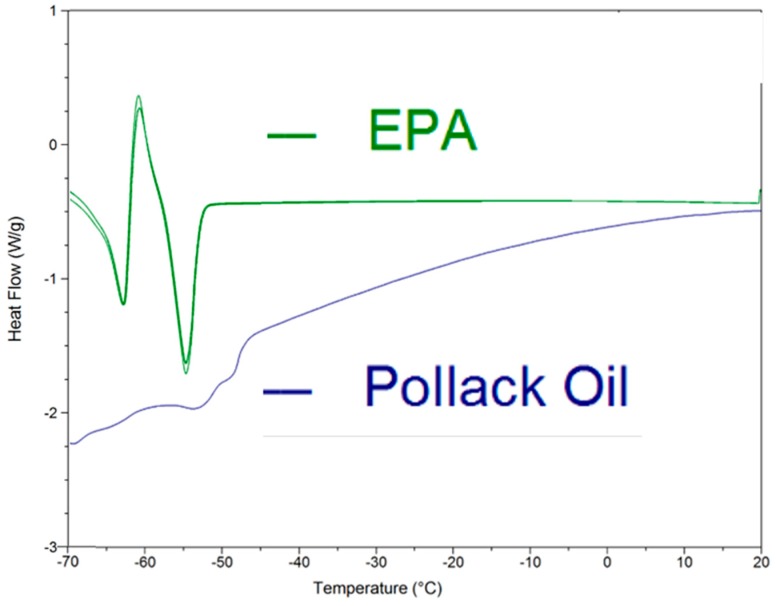
Linear ramp differential scanning calorimetry heat absorption data at 2 °C min^−1^ for eicosapentaenoic acid (EPA) and 4 °C min^−1^ and pollack oil (PO). From −70 to −50 °C EPA shows endothermic-exothermic-endothermic pattern characteristic of a solid state phase transition followed by melting. The reversal curve for EPA shows that the pattern occurs with cooling as well as heating. PO absorbs heat gradually and melts within the −65 to −45 °C range.

**Figure 2:**
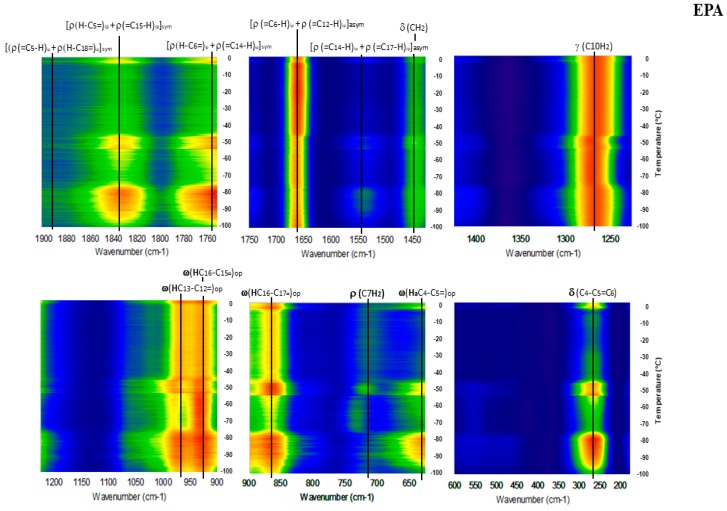
EPA gradient temperature Raman spectroscopy (GTRS) contour plot with vibrational mode assignments. Intensity normalized to 1650 cm^−1^. Note, major Raman mode discontinuities and frequency shifts that correlate with the large changes in heat flow shown in Figure 1.

**Figure 3:**
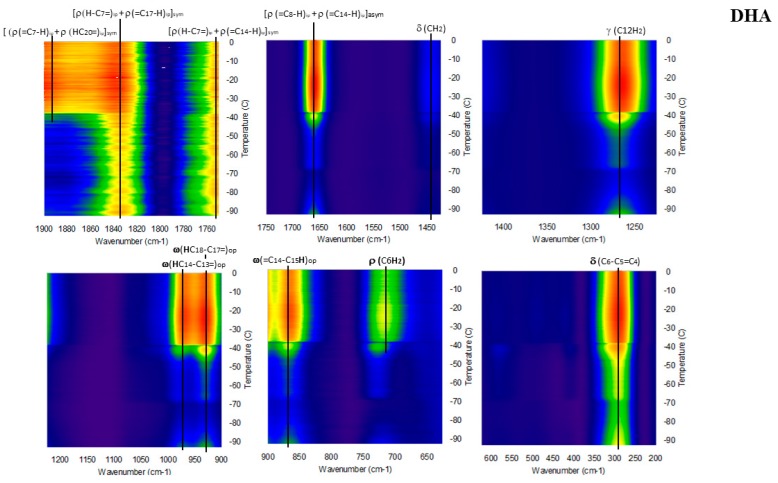
DHA GTRS contour plot with vibrational mode assignments. Melting is complete and heat flow returns to baseline at −40 °C [17].

**Figure 4:**
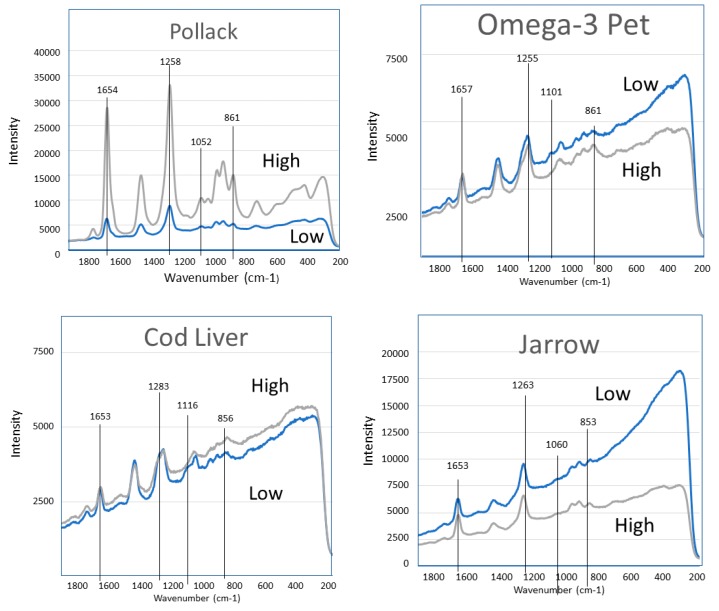

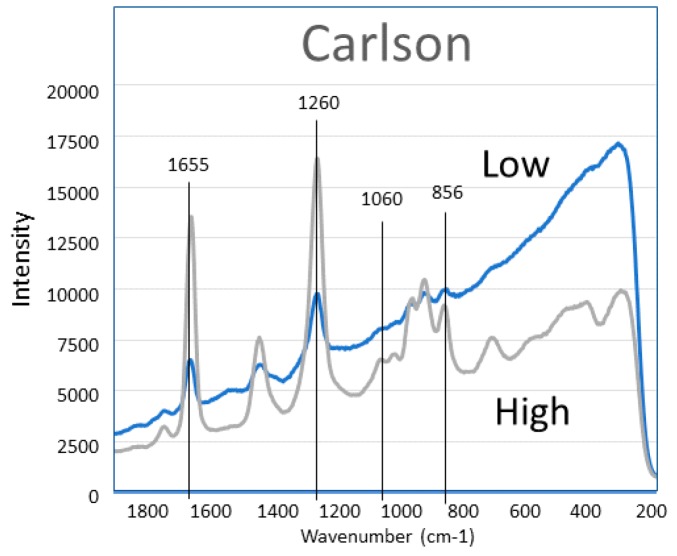
Representative raw line spectra for each sample when all liquid (18 °C; high) and all solid (−70 °C; low).

Figure 1 gives DSC data for EPA, which shows a clear, reversible pattern indicative of a dramatic solid state phase transition, followed by melting centered at −55 °C and complete by −50 °C. This distinctive pattern mirrors that of oleic acid (OA; 18:1n-9), which has a reversible transition from the γ to α solid phase at −2.2 °C followed by melting at 13.3 °C [18,27]. Figure 2 shows the GTRS contour plot. Strong shifts in intensity—including modes that disappear and reappear—as well as complex frequency shifts—correlate with the DSC heat flow data. Vibrational mode assignments are given in Table 1 along with data for DHA for comparison. Derivative spectra are utilized to aid in assignments and are available on request. Figure 3 shows the contour plot for DHA. Note that the contour plots are created by stacking hundreds of single temperature spectra, each of which is individually retrievable with the software. Figure 4 gives a representative overview of the line spectra. 

**Figure 5:**
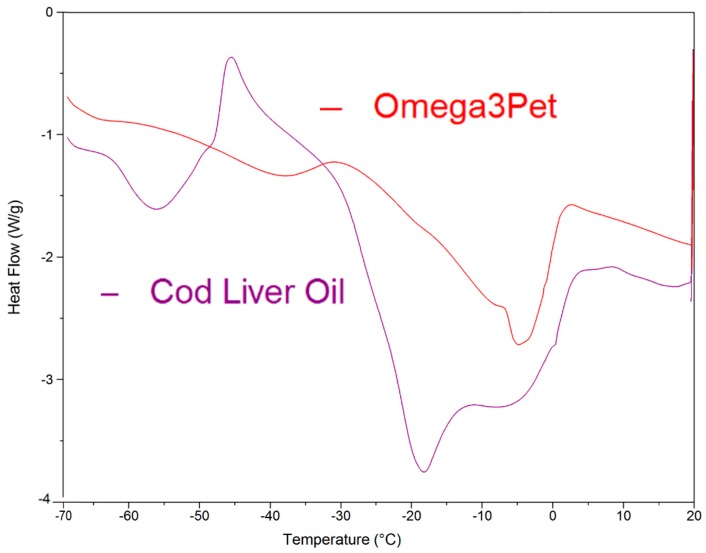
Linear ramp differential scanning calorimetry heat absorption data at 4 °C min^−1^ for cod liver oil (CLO) and Omega-3 Pet oil (O3P; specified as anchovy and sardine).

Additionally, given in Figure 1 are DSC data for pollack oil (PO), which shows a broad area of solid state rearrangement followed by melting, which is complete by −40 °C. The heat flow gradient is much shallower and less distinct than that of the EPA. The Jarrow (JW) and Carlson (CN) fish oils had similar, shallow heat flow curves (not shown). Figure 5 shows the DSC heat flow curves for Omega 3 Pet fish oil (O3P) and cod liver oil (CLO). CLO has a pronounced exothermic region from −55 to −35 °C and a pronounced endothermic area peaking at −18 °C, indicating melting is complete. O3P shows a similar exothermic/endothermic pattern with melting complete circa −5 °C.

**Figure 6:**
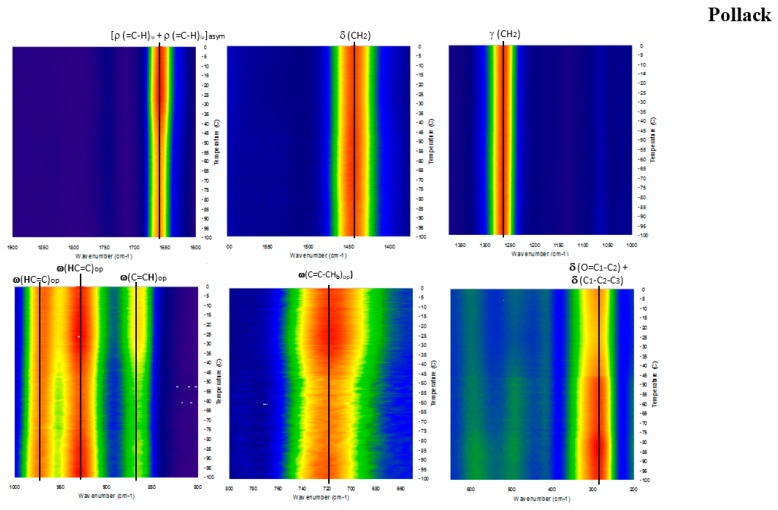
GTRS contour plot, Alaskan pollack oil concentrate (PO). Note, minor Raman mode intensity change and line broadening that correlate with heat flow data in Figure 1.

**Figure 7:**
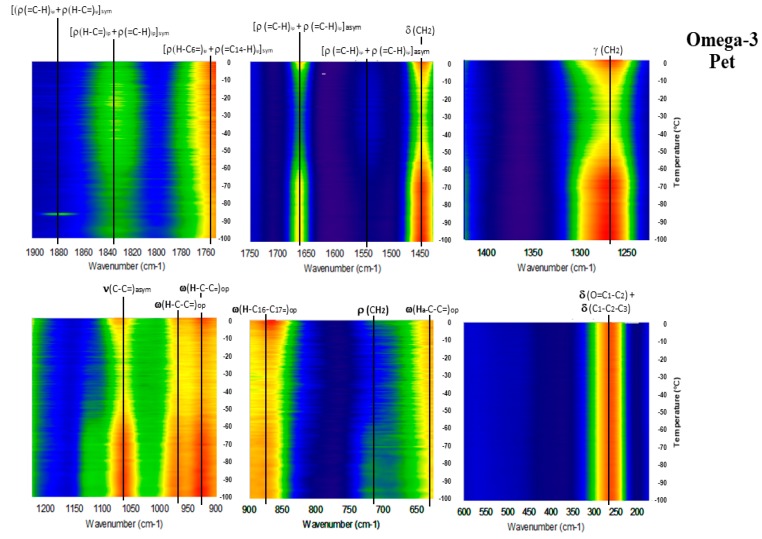
GTRS contour plot, Anchovy and sardine oil (O3P). Note, major Raman mode intensity changes and discontinuities that correlate with heat flow data in Figure 5.

**Figure 8:**
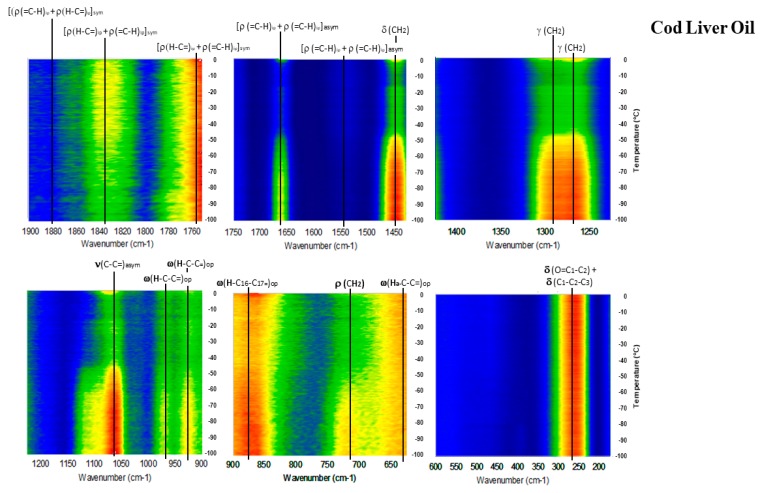
GTRS contour plot, Arctic cod liver oil (CLO). Note, major Raman mode intensity changes and discontinuities that correlate with heat flow data in Figure 5.

**Figure 9:**
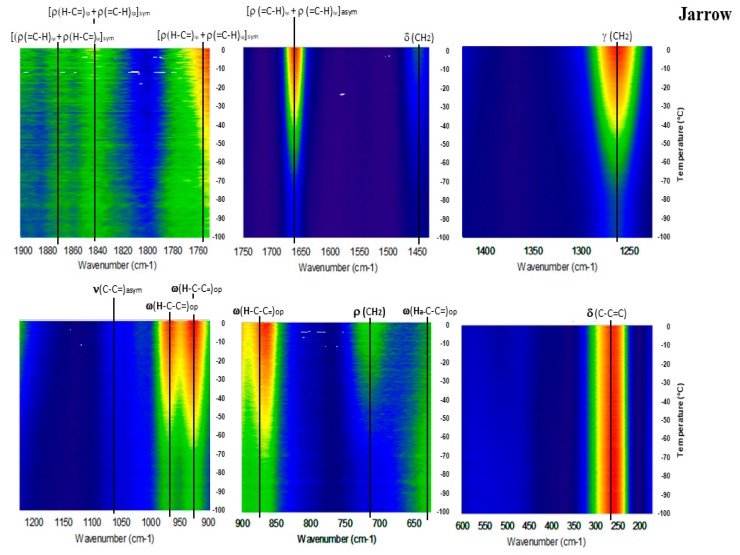
GTRS contour plot, fish oil concentrated in DHA by molecular distillation (JW).

**Figure 10:**
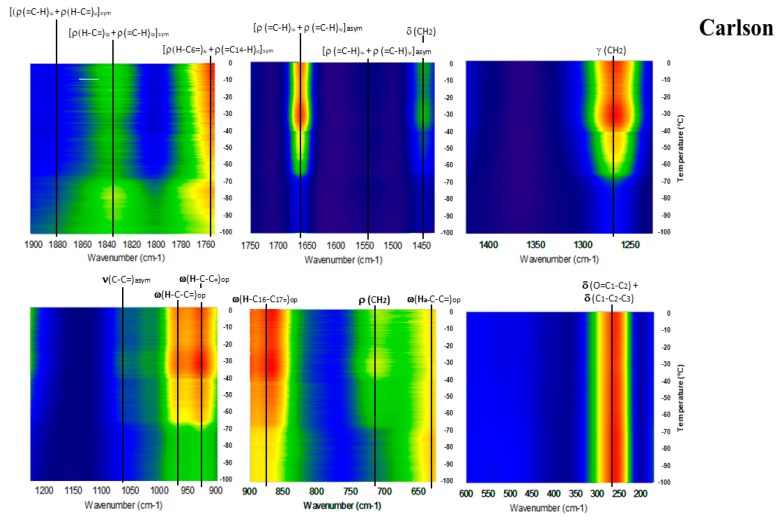
GTRS contour plot, Norwegian fish oil concentrate from anchovy, sardine, mackerel (CN).

Figure 6, Figure 7, Figure 8, Figure 9 and Figure 10 show the GTRS contour plots for each product from cryogenic temperatures to 0 °C. Some additional data above 0 °C are given in the Supplementary section. Other than a lemon scent added to CN, none of the five can be clearly distinguished on the basis of smell or appearance, yet each has a distinct, individualized Raman spectrum within a temperature gradient. While the individual line spectra have some distinctions, they are not as diagnostic as the contour plots which trace the spectroscopy through solid, melting, and liquid. The Raman spectra are particularly distinctive when solid−solid or solid−liquid transitions are passed. As temperature increases, the spectra broaden and simplify, and each oil cannot be distinguished graphically as they can with the contour plots. 

Pollack Oil (PO; Figure 6 and Figure S1): The data are particularly sharp and clean for this sample, in agreement with the stated >90% triacylglycerol (TAG) content. Broadening and shifting of modes is indicative of melting, and is complete circa −45 °C, which is also the inflection point on the DSC when melting is complete. Beyond this point heat flow increases smoothly, and there is very little spectral change. The product label specifies zero saturated fat. Strong, broad and constant intensity from CH_2_ scissoring (symmetric H rocking motion perpendicular to the plane of C-C) circa 1440 cm^−1^ across the temperature range is indicative of OA, but can also include some saturated fat. This mode also exists in linoleic acid (18:2n-6), n-3DPA and EPA but is weaker and has greater temperature dependence. Notably the 1050 to 1150 cm^−1^ modes are very weak; they are barely visible in Figure 6 but resolved in the derivative plots and in the 0 to 20 °C spectrum (Figure S1). These modes are C-C and C-C=C symmetric and asymmetric stretching. In the case of Figure 6, only asymmetric modes are present, which effectively cancel out and reduce intensity. 

Omega-3 Pet (O3P; Figure 7 and Figure S2) also shows high intensity in the solid cryogenic region, followed by minimum intensities in the endothermic region of the heat flow. Intensities for most modes begin to increase again circa −5 °C, the minimum of endothermic region, indicating melting is complete. In the 0 to 20 °C spectrum, 1440 cm^−1^ intensifies strongly, indicative of saturated fatty acids, and concurrently 1830 cm^−1^ weakens. Distinct modes at 875, 925 and 975 cm^−1^ merge into a single broad peak. The 1050 to 1150 cm^−1^ modes are present in the cryogenic range with greatest intensity at 1075 cm^−1^ (symmetric). Considerable asymmetric stretching is seen in the solid phase but decreases as temperature increases. In the 0 to 20 °C spectrum (Figure S2) the mode is strongly symmetric. The spectrum required some fluorescence correction, probably reflecting some percentage phospholipid in the product. 

Cod Liver Oil (CLO: Figure 8 and Figure S3) shows high intensity in the solid cryogenic region for all modes, but most modes dramatically weaken circa −45 °C, which is at the maximum of the DSC exothermic peaks. Intensity remains low until −18 °C, the minimum of the endothermic region of the heat flow, indicating melting is complete. The 0 to 20 °C spectrum (Figure S3) is similar to O3P, with 1440 cm^−1^ strongly intensifying and 1830 cm^−1^ concurrently weakening. The 1050 to 1150 cm^−1^ mode pattern described for O3P also appears in this sample. The spectrum required some fluorescence correction, probably reflecting some percentage phospholipid in the product. 

Jarrow Max DHA (JW; Figure 9): This product has the highest DHA percentage of all products analyzed. In contrast to PO, O3P and CLO, this sample has steadily increasing intensity with temperature. Further, 1075 and 1440 cm^−1^ are virtually absent, and 715 cm^−1^ is very weak until after −40 °C, when the sample has melted. High frequency modes up to 1900 cm^−1^ show moderate intensity throughout the temperature range. All of these spectral features are similar to pure DHA as a free fatty acid (Figure 3 [17,19]).

Carlson FO (CN; Figure 10 and Figure S4) had the highest percentage of EPA of all products analyzed. Intensity increases with temperature but not as smoothly as for JW. There is a distinct intensity increase circa −70 °C, followed by a very strong increase in intensity circa −40 °C, where the sample has melted. Additionally, 1075 and 1450 cm^−1^ are weak but not absent, and 715 cm^−1^ is present throughout the temperature range.

## 3. Discussion

GTRS provides a new means for rapid high throughput material identification and quality analysis. The entire set or any subset of the contour plots can be utilized to create a graphical standard without background matching, peak subtraction, or principal component analysis (PCA) to quickly authenticate a given source. The test dataset is simply graphically overlaid on the first set. The contour plots, first derivatives and second derivatives can all be utilized graphically. In addition, for any given sample, a temperature range can be specified that maximizes the information content. For example, −85 to −80 °C might not be of special interest for JN or PO, but is very interesting for CLO and O3P, because the latter two samples show intense solid state vibrations in that range which are highly temperature dependent. Compared to JN and PO, CLO and O3P have very different solid state structures that continue to absorb heat and melt over a large temperature range. In those ranges there will be a mixture of liquid and solid phases, which changes the nature of the Raman signal. Omega-3 Pet, specified as anchovy and sardine oil, is also the sample with the highest melting temperature, yet is liquid at the lowest temperature where marine fish exist (−2 to −3 °C, depending on salinity). Zhang et al. [25] found over 20 TAG species in anchovy, salmon and tuna oil, with each fish differing significantly. Anchovy oil in particular was 48% myristic (14:0) and palmitic (16:0) acid, but only three percent of the identified TAG were fully saturated. Tripalmitin and trimyristin have melting ranges that start at 45 and 56 °C respectively, and if a significant fraction of the 48% existed in those forms the melting temperature and GTRS contour plots will differ significantly, as would North Atlantic fish biology.

Even in the readily technically achievable gradient of −20 to 20 °C there is still distinctive spectroscopy among the five fish oil samples as compared to a single room temperature Raman or FTIR spectrum. As mentioned in the Introduction, if contour plot matching determines that a sample varies from the standard beyond the specified limits, more precise but also more expensive and time consuming analyses can be performed. Clearly quantitative lipid chromatographic analyses cannot be replaced, but they can be both reduced in number and complemented with both conventional IR and Raman [13,20,22] and GTRS. Deconstructive analyses can identify percentages of fatty acids, and with very detailed work TAG, but they cannot necessarily quickly identify unlabeled cod liver, tuna, pollack, salmon and other marine oils. With appropriate standardization, GTRS has the potential to rapidly and nondestructively identify individual fish and many other edible oils. Modifications can allow analysis of encapsulated oils, either directly through the capsule [15] or by offset analysis, which utilizes a specific beam angle to analyze content inside a capsule [28]. 

Although for some applications GTRS may obviate the necessity for PCA, in the event that it is desired GTRS can improve the analysis by adding components and the extra dimension of temperature. For example, previous work on adulteration of cod liver oils with animal fats found that the 25 °C FTIR spectra for chicken, beef, mutton, pork and cod liver oil were nearly identical [29,30]. Only relatively minor changes in the 1030 cm^−1^ region were utilized to model 0.5% to 50% lard mixed into cod liver oil. 

Domestic animal fats are uniformly rich in TAGs composed of stearic, palmitic and oleic acids, with a much higher percentage of fully saturated and disaturated/monounsaturated species than fish. When these are mixed into fish oil, the overall intensities of the 1440 and 1075 cm^−1^ modes will increase, and the temperature dependence of their respective intensities will also change. A third definitive spectral region such as the circa 720 cm^−1^ region can be selected, and the relative intensities of all modes considered concurrently at, for example, four selected temperatures or within a given temperature range. This can yield improved component separation as compared to a single mode at a single temperature. 

It must also be acknowledged that mixed lipids interact significantly, mixing nonideally in the liquid phase and separating into multiple solid phases as they cool [18,31,32,33]. Consequently, their spectroscopy can change far more than just a dilution factor as is the assumption with models such as [30]. Lipids rich in n-3 (or n-6) PUFA will have some moieties in common that will likely pack fairly uniformly and similarly; but in other locations, unavoidable lipid chain dissimilarities will create sites of increased elasticity, torsion and twisting. While such interactive mixing behavior is undoubtedly complex and difficult to fully quantify in a natural highly polyunsaturated lipid, GTRS allows one to utilize it in a qualitative sense. Lard or tallow added to cod liver oil for purposes of economic adulteration (at least 10%) will change the melting behavior enough that it can be readily identified by GTRS, DSC or even simpler methods. A physical mixture of highly saturated TAGs and highly polyunsaturated TAGs responds to a thermal gradient very differently than a TAG containing a mixture of saturated and polyunsaturated fatty acids. 

Similarly, consider PO, the sample which showed the least temperature dependence across the spectrum. It is likely this sample is composed of a limited number of isomers. Melting can be still identified, particularly in the broad out-of-plane bending modes [ω(C=C-CH**_b_**)_op_] centered at 725 cm^−1^. However, these modes are not present in saturated fats and one only one mode [731 cm^−1^ ω(=CH10-C11HaHb)] is present in in OA and it does not change until −4 °C [18]. Adulteration of this oil with animal or vegetable fats, either in TAG or phospholipid form, will change the Raman spectroscopy in this key region when examined in a temperature gradient.

### Detailed Spectroscopy of EPA and DHA

The commercial fish oils investigated are marketed mainly on the basis of providing supplemental EPA and DHA. Although EPA is the precursor to DHA, provision of both LC-PUFA is considered valuable due to the poor conversion rate of EPA to DHA and the functional biochemical differences between the two [5,34,35,36]. GTRS analysis in the simpler systems of pure free fatty acids can identify torsion at specific molecular sites, which is a marker of discrete differences in lipid conformation. Each of eight unsaturated fatty acids we have investigated has a unique three-dimensional conformation that is driven by the orientation of CH or CH_2_ groups within the carbon backbone. Further, each of these lipid conformations responds to a temperature gradient in a different manner. This is the basis for the contour plots’ ability to create unique graphical standards: Complex lipids with a variety of fatty acids and isomers also have three dimensional structures incorporating torsion, and in addition they must pack, especially in the solid state. 

EPA has a distinctive endothermic/exothermic/endothermic DSC heat flow curve with relatively large changes in heat flow. DHA and n-3DPA also showed this complex endothermic/exothermic pattern followed by a strong endothermic melting peak in DSC runs at 1 or 2 °C min^−1^. However, this pattern was not seen at 10 °C min^−1^, and the heat flow changes prior to melting were minor in magnitude compared to fusion [17,19]. Nonetheless, they were correlated to premelting changes in the GTRS contour plots; some of these changes can be observed in Figure 3, but overall the DHA Raman spectra have less temperature dependence than those of EPA. As mentioned previously, the EPA DSC data bear resemblance to the well-quantified phase transitions of OA (γ to α solid phase at −2.2 °C; α melting at 13.3 °C [27]), which strongly correlate with distinctive changes in the Raman spectra [18]. The arachidonic acid (AA; 20:4n-6) DSC data are similar: A large exothermic peak from −60 to −80 °C that may represent crystallization was observed prior to melting (−37 °C), and the DSC thermogram was correlated to complex and dramatic changes in the GTRS spectra [20]. 

Suzuki et al. [37] reported the temperature-dependent enthalpy (ΔH) of the OA γ to α transition and α fusion as 8.76 kJmol^−1^ and 39.6 kJmol^−1^ respectively. Our DSC analysis for OA is in agreement with these authors, which allows for estimation of the magnitude of the energetic changes observed in the DSC data for EPA and AA. Peak-to-peak ΔH (integrating through the baseline) for the complex endothermic/exothermic peak seen in EPA is 43 kJmol^−1^ and peak-to-peak melting is 30 kJmol^−1.^ For AA, the exothermic peak ΔH is estimated at 18 kJmol^−1^; in this case the heat flow returns to baseline, followed by melting (ΔH~29 kJmol^−1^). In contrast, Ueno et al. [38] reported two solid state phase transitions for LA at −51.3 °C (ΔH = 2.6 kJmol^−1^) and −33.5 °C (ΔH =0.27 kJmol^−1^) followed by melting at −5.8 °C. We only observed one (−51.3 °C) solid state phase transition with DSC, and neither temperature region showed significant GTRS spectral changes [18]. Overall, phase changes or molecular rearrangements that are fairly energetic are able to affect molecular polarizability enough that they significantly change the Raman signal. When observed in a temperature gradient, even transient Raman changes can be recorded and may be as distinctive and useful as DSC data.

Interestingly, the spectroscopy of EPA is more similar to that of DHA than either of the DPAs [17,19], indicating that the maximization of methylene groups in both of these structures is more important than the 22-carbon chain length. The moiety C20 to C4 in EPA is the same moiety as C22 to C6 in DHA; in these regions the structures, conformations, and changes in conformation with temperature are very similar. Differences exist in the carbonyl end, where EPA contains C4H_2_-C3H_2_-C2H_2_-COOH but DHA has (H-C5=C4-H)-C3H_2_-C2H_2_-COOH—an additional double bond and but one less methylene group. In DHA all seven CH_2_ moieties adjacent to the double bonds are on the concave curvature of C=C bonds, including those at C6 and C3, but site C2 is on the convex side of C=C curvature. In EPA, all six CH_2_ sites adjacent to the double bonds are also on the concave curvature of C=C sites, including C2. This results in near zero CH_2_ rocking at C6 in EPA but very strong C6 rocking in DHA. In EPA however, C3 (no longer adjacent to a double bond) is on the convex curvature side, and is responsible for the somewhat stronger 1440 cm^−1^ CH_2_ scissoring in EPA vs. DHA; in the latter molecule this mode is weak because it only originates from the methyl group. 

All vibrational modes in the spectral region from 1900 to about 1475 cm^−1^ involve C=C stretching. Assuming all carbon-carbon double bond stretching was equal, only a single vibrational mode (near 1655 cm^−1^) would be observed. Instead, for all PUFA except LA three or more strong C=C stretching modes are observed, and are “fingerprints” that distinguish differences in structure and conformation in among C-C= and C=C-C sites. The stretching is conformational and temperature dependent in part because for *cis*- H atoms on C=C sites, H-C= rocking does not necessarily equal =C-H rocking. In this spectral region EPA and DHA have four common vibrational modes: 1892, 1832, 1754 and 1662 cm^−1^. For DHA above −40 °C, the relative intensities of 1832 cm^−1^ ([ρ(=C_7_-H) + ρ(H-C_17_=)]) and 1892 cm^−1^ ([ρ(=C_7_-H) + ρ(H-C_20_=)]) are similar and the region becomes broadened to the point that it is nondiagnostic above 0 °C. However, below −40 °C 1832 cm^−1^ is dominant and may be the only mode present, indicating that the methyl end is less rigid and more able to absorb thermal energy. An additional low intensity peak near 1540 cm^−1^ is observed in EPA between −90 and −80 °C. Assignable to ([ρ(=C_14_-H) + [ρ(=C_17_-H)]), this vibrational mode enables the C14=C15 and C17=C18 double bonds to remain co-planar. Unless these two sites are sufficiently co-planar, a conformation that allows the vibrational mode at 1892 cm^−1^ to occur and be observed would not be possible.

In conclusion, lipids have complex three dimensional structures which have individualized responses to a thermal gradient that are detectable with Raman spectroscopy. This behavior is useful not only for theoretical investigations of molecular structure and dynamics, but also for practical, inexpensive and rapid product authentication. Large increase in overall Raman intensity, line broadening and some frequency shifts are correlated with melting. Energetic solid state phase transitions and premelting transitions can be correlated with frequency shifts, intensity changes, and with vibrational modes that appear or disappear (or nearly so). Even when the phase equilibria of the lipid are unknown, or the material is a complex lipid, these principles apply to identify suspected transitions and to guide further research in the right direction. 

## 4. Materials and Methods

Sample source and description are as follows, including listed DHA, EPA and other omega-3 concentrations per 1000 mg. Materials were stored sealed in a freezer or refrigerator until immediately prior to use

- *Cis*-5,8,11,14,17 Eicosapentaenoic acid 98%, Sigma-Aldrich Inc., St. Louis, MO, USA. (EPA)
- Wiley’s Finest Alaskan Pollack oil concentrate was donated by Wiley’s Finest Inc. Coshocton, OH, USA. Liquid form, specified as >90% TAG. DHA 189 mg; EPA 289 mg; other omega-3 56 mg. (PO)
- Omega-3 Pet, Nordic Naturals, Inc. Watsonville, CA, USA; purchased from a retail vendor. Anchovy and sardine oil, liquid form, specified as TAG. DHA 100 mg; EPA 170 mg; other omega-3 40 mg. (O3P)
- Pet Cod Liver Oil, Nordic Naturals, Inc. Watsonville, CA, USA; purchased from a retail vendor. Wild Arctic Cod liver oil, liquid form, specified as TAG. DHA 120 mg; EPA 80 mg; other omega-3 50 mg. (CLO).
- Max DHA fish oil, purchased directly from Jarrow Formulas, Inc. Los Angeles, CA, USA. Encapsulated fish oil concentrated in DHA by molecular distillation. Unspecified fish source. DHA 600 mg; EPA and other omega-3 not listed but is within range 50–250 mg. (JW).
- Carlson Maximum Omega 2000 fish oil was donated by J.R. Carlson Laboratories, Arlington Heights, IL, USA. Encapsulated Norwegian fish oil concentrate from anchovy, sardine, mackerel. Reported (but not specified) as TAG. DHA 190 mg; EPA 480 mg; other omega-3 96 mg. (CN).

The GTRS system utilizes a 785-nm laser module (I0785MM0350MF-NL, Innovative Photonic Solutions, Monmouth Junction, NJ, USA) as the excitation source. A fiber optic Raman probe (RPB, InPhotonics, Norwood, MA, USA) is used to focus the laser and acquire the Raman signals. A bifurcated fiber bundle with a 16-bit CCD camera (1024 × 256 pixels; Newton DU920N-BR-DD, Andor Technology, South Windsor, CT, USA) delivers the laser radiation to the probe and transmits the Raman signals to the spectrometer. The spectrometer detects a Raman shift range of 102.2 to 2538.1 cm^−1^ with a minimum spectral resolution of 3.7 cm^−1^. 

Samples were placed in aluminum pans which were then placed on large copper heat sinks with a sample platform on the upper surface. The heat sink and sample holder were chilled in a LN_2_ bath, but the sample itself did not come in direct contact with LN_2_ or atmospheric water vapor condensate. A ceramic hotplate was used for controlled heating of samples and as a stable insulating platform for analysis. The copper heat sinks were placed directly on the ceramic heat surface and served to buffer the rate of temperature increase. Two K-type thermocouple probes (range −200 to 1350 °C) are attached to two sides of the sample area and connected to a dual-input thermometer (EasyView EA15, Extech Instruments, Nashua, NH, USA). The sample temperature was defined as the average value of the two probes. The thermocouple calibration based on test resistance provided by the manufacturer was utilized as well as calibration in LN_2_. The Raman probe, hotplate, and sample materials were placed in a closed black box to avoid ambient light. Raman spectra can be acquired from −180 to 320 °C. The heating gradient was approximately 1 °C min^−1^ for these mainly cryogenic investigations. We did not use a set time schedule for spectral acquisition, but rather acquired spectra each time the sample temperature increased either 1 or 0.2 °C, depending on the analysis. System software was developed using LabVIEW 2018 (National Instruments, Austin, TX, USA) to fulfil functions such as camera control, data acquisition, temperature measurement, signal/noise threshold and synchronization. All Raman samples were run in triplicate and each dataset analyzed independently to ensure reproducibility.

SigmaPlot 13 (Systat Software, Inc., San Jose, CA, USA) generated three-dimensional contour plots (frequency, temperature, signal intensity). The contour plots were created by stacking hundreds of single temperature spectra, each of which was individually retrievable with the software. Weaker and/or overlapping modes that are not obvious on the contour plots were resolved within these spectra, or were observable in the derivative plots. Some data required standard fluorescence correction. Software counting statistics ensured that only data with 99% certainty of true signal were utilized. First derivative intensity contour plots were calculated using a three-point running average, i.e., the first and last points calculated the slope of the middle point. The contour plots for the second derivative intensity were calculated using the three-point running average data from the first derivative spectra. Relative intensity for fish oils was normalized to 300 cm^−1^, which had minimum temperature dependence and was below the major region of interest. For EPA and DHA relative intensity was scaled normalized with the very intense C=C stretch region circa 1650 cm^−1^. Maximum to minimum intensity colors were red > orange > yellow > green > blue > black. Each contour data array contained about 20 Mb. 

For DSC data, 20 mg of lipid sample equilibrated to 25 °C was scanned in a crimp-sealed aluminum sample pan. We utilized a Q200 differential scanning calorimeter (TA Instruments, New Castle, DE, USA) with heating rates of 4.0 °C min^−1^ for fish oils and 2.0 °C min^−1^ for EPA from −80 to 20 °C. Samples were scanned under a continuous N_2_ flow heated at the same rate as the sample. Samples for both instruments were run in triplicate, and each dataset analyzed independently to ensure reproducibility.

## Figures and Tables

**Table 1:** EPA and docosahexaenoic acid (DHA) modes and vibrational assignments; a, b, c are carbon numbers from methyl end. Original DHA data from ref. [17].

DHA (cm^−1^)		EPA (cm^−1^)		Spectral Assignments		
−100 to −50 °C	−50 to −10 °C	−100 to −30 °C	−50 to −10 °C			
					DHA _a,b_	EPA _a,b_
	1851	1849	1849	[ρ(H-C_a_=) + ρ(=C_b_-H)]_sym_	_7,17_	_5,15_
	1784	1782	1782	[ρ(=C_a_-H) + ρ(H-C_b_=)]_sym_	_8,16_	_6,14_
	1759	1757	1757	[ρ(H-C_a_=) + ρ(=C_b_-H)]_sym_	_7,14_	_5,12_
					DHA _a,b_	EPA _a,b_
1698	1698	1698	1698	[ρ(H-C_a_=) + ρ(=C_b_-H)]_asym_	_10,16_	_8,14_
1644	1644	1647	1647	[ρ(=C_a_-H) + ρ(H-C_b_=)]_asym_	_8,14_	_6,12_
	1565	1568	1568	[ρ(=C_a_-H) + ρ(H-C_b_=)]_asym_	_16,19_	_14,17_
1478	1478	1481	1481	[ρ(H-C_a_=) + ρ(=C_b_-H)]_asym_	_10,13_	_8,11_
1437	1437	1437	1437	δ(CH_2_)		
					DHA _a_	EPA _a_
	1408	1392		δ(C_a_H_2_)	_9_	_7_
1341	1341	1338	1338	γ(C_a_H_2_)	_12_	_10_
1263		1263	1263	γ(C_a_H_2_)	_9_	_7_
1234	1234	1235	1235	γ(C_a_H_2_)	_15_	_13_
					DHA _a,b_	EPA _a,b_
1094	1094	1088	1088	ν(C_a_-C_b_=)_asym_	_21,20_	_19,18_
1049	1049			ν(C_a_-C_b_=)_asym_	_3,4_	
972		972		ω(H-C_a_-C_b_=)_op_	_12,11_	_10,9_
	958	958	958	ρ(C_a_H_2_)	_9_	_7_
914	914	914	914	ω(H-C_a_-C_b_=)_op_	_6,7_	_4,5_
					DHA _a,b_	EPA _a,b_
851	851	851	851	ω(=C_a_-C_b_H_α_)_op_	_14,15_	_12,13_
	827	827		ν(C_a_-C_b_=)_asym_	_18,19_	_16,17_
754	754	754	754	ρ(C_a_H_2_)	_12_	_12,11_
715		715		ω(H_α_C_a_-C_b_=)_op_	_6,5_	_4,5_
	696	696	696	ρ(C_a_H_2_)	_6_	_4_
651				ω(H-C_5_=C_6_)_op_	_3,4_	_4_
					DHA _a,b,c_	EPA _a,b,c_
563		544		δ(C_a_-C_b_=C_c_) + δ(C_a_=C_b_-C_c_)	_12,11,10 + 8,7,6_	_10,9,8 + 6,5,4_
464	464	473	473	δ(C_a_C_b_C_c_)	_2,3,4_	_2,3,4_
	415	406	406	δ(O=C_a_-C_b_) + δ(C_a_-C_b_=C_c_)	_1,2 + 1,2,3_	_1,2 + 1,2,3_
265	265	252	252	δ(C_a_-C_b_=C_c_)	_3,4,5_	_4,5,6_

## Supplementary Materials

Four supplemental figures (Figures S1–S4) providing additional data above 0 °C. The supplemental materials are available online.
