# Supplementary material for: Continuous Gradient Temperature Raman Spectroscopy of Fish Oils Provides Detailed Vibrational Analysis and Rapid, Nondestructive Graphical Product Authentication
Source: Molecules. 2018 Dec 12;23(12):3293. doi: 10.3390/molecules23123293 (PMC6320940; doi:10.3390/molecules23123293)
Supplement: Supplementary file 1 [file molecules-23-03293-s001.pdf]

Supplementary Materials

Continuous Gradient Temperature Raman Spectroscopy of Fish Oils Provides Detailed Vibrational Analysis and Rapid, Nondestructive Graphical Product Authentication

C. Leigh Broadhurst [1,2,*], Walter F. Schmidt [1], Jianwei Qin [1], Kuanglin Chao [1] and Moon S. Kim [1]

[1] Sensors Development Laboratory, Environmental Microbiology and Food Safety Laboratory, United States Department of Agriculture Agricultural Research Service, 10300 Baltimore Avenue, Beltsville, MD 20705, USA; walter.schmidt@ars.usda.gov (W.F.S.); jianwei.qin@ars.usda.gov (J.Q.); kevin.chao@ars.usda.gov (K.C.); moon.kim@ars.usda.gov (M.S.K.)

[2] Department of Mechanical Engineering, University of Maryland Baltimore County, Baltimore, MD 21250, USA

* Correspondence: leigh.broadhurst@ars.usda.gov; Tel.: +1-301-504-8450 x237

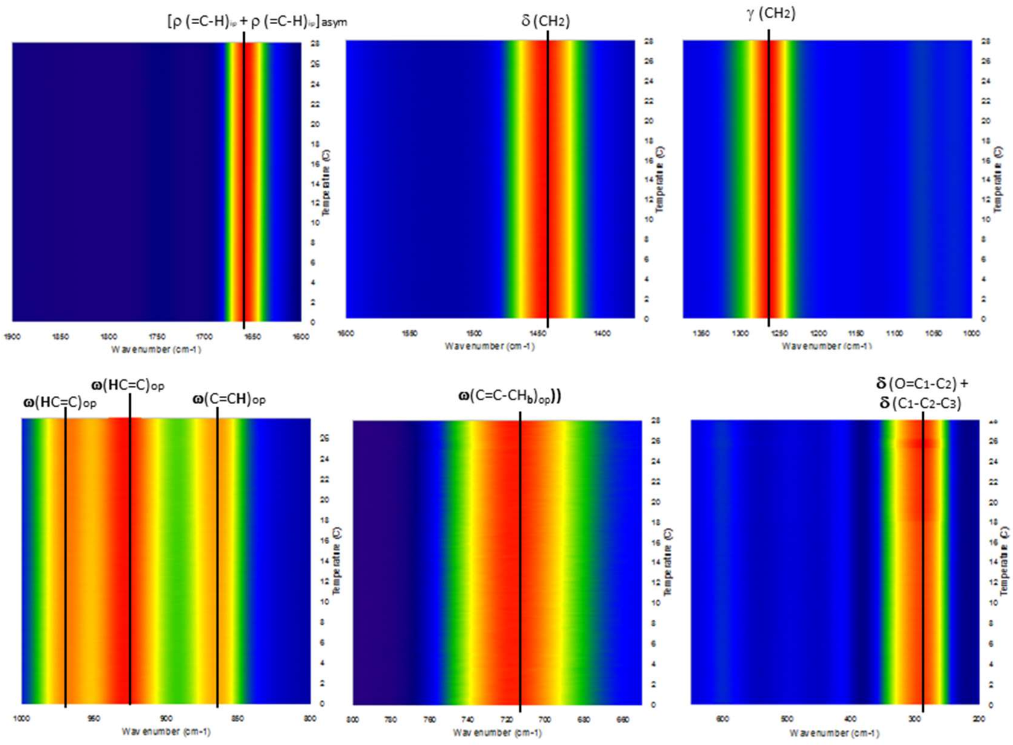

Figure S1. Pollack Oil 0 to 28 °C

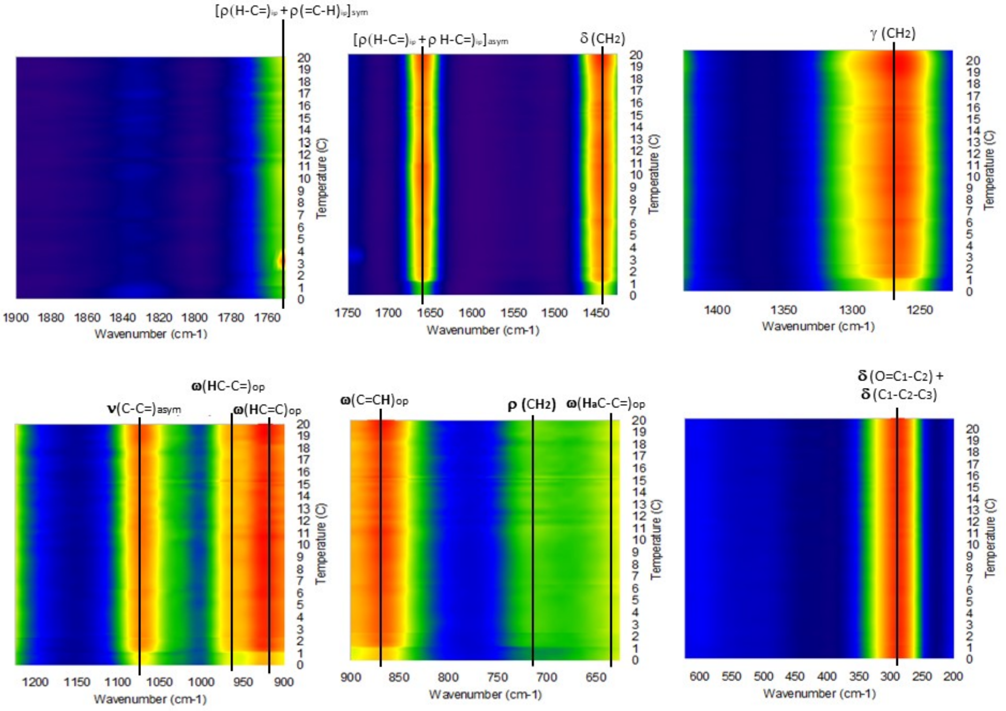

Figure S2. Omega 3 Pet 0 to 20 °C.

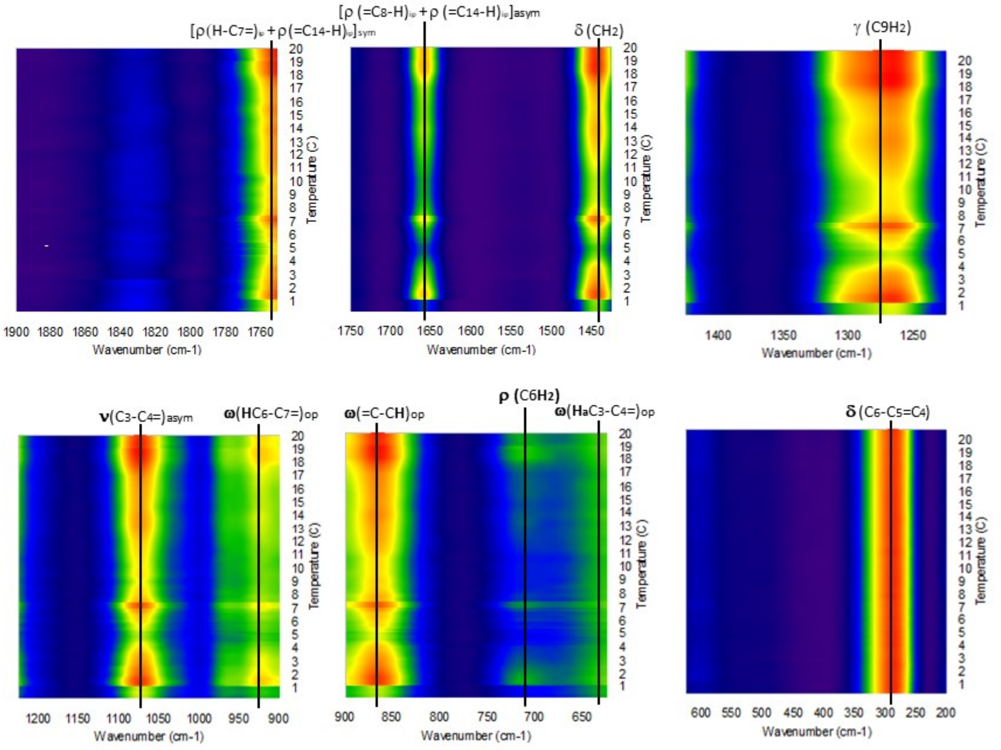

Figure S3. Cod Liver Oil 0 to 20 °C.

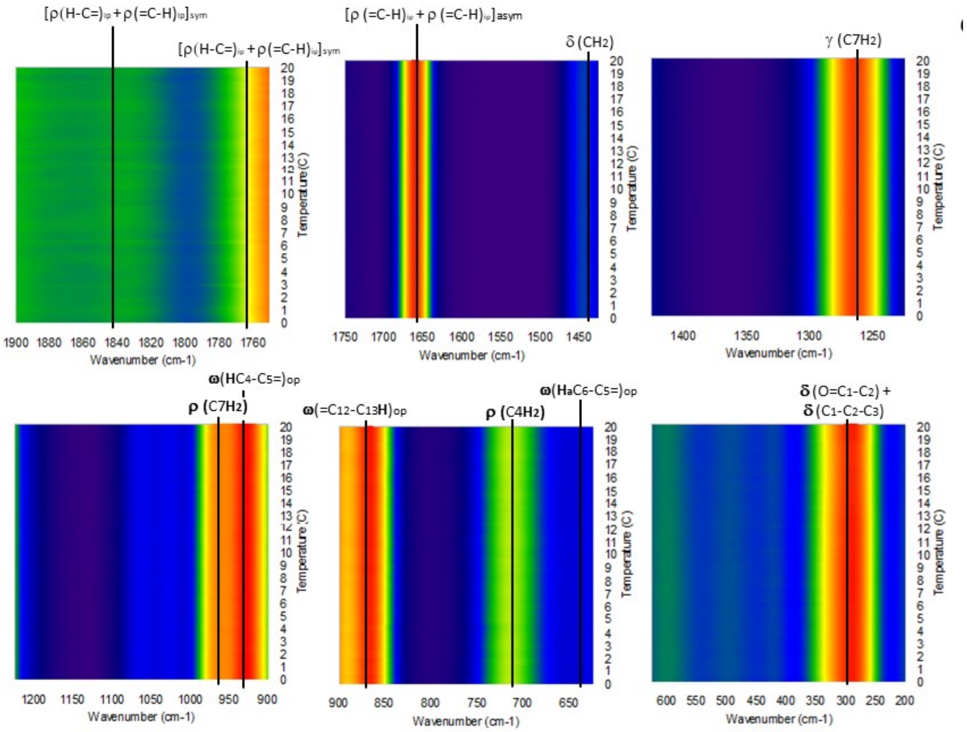

Figure S4. Carlson Fish Oil 0 to 20 °C.
